# The Multiple Functions of Common Microbial Carbon Polymers, Glycogen and PHB, during Stress Responses in the Non-Diazotrophic Cyanobacterium *Synechocystis* sp. PCC 6803

**DOI:** 10.3389/fmicb.2016.00966

**Published:** 2016-06-21

**Authors:** Ramon Damrow, Iris Maldener, Yvonne Zilliges

**Affiliations:** ^1^Section of Plant Biochemistry, Institute of Biology, Humboldt-Universität zu BerlinBerlin, Germany; ^2^Section of Organismic Interactions, Interfaculty Institute of Microbiology and Infection Medicine, Eberhard Karls Universität TübingenTübingen, Germany; ^3^Section of Biophysics and Photosynthesis, Institute of Physics, Freie Universität BerlinBerlin, Germany

**Keywords:** glycogen, PHB, metabolic spilling, cyanobacteria, nitrogen chlorosis, thylakoid disassembly

## Abstract

Classical microbial carbon polymers such as glycogen and polyhydroxybutyrate (PHB) have a crucial impact as both a sink and a reserve under macronutrient stress conditions. Most microbial species exclusively synthesize and degrade either glycogen or PHB. A few bacteria such as the phototrophic model organism *Synechocystis* sp. PCC 6803 surprisingly produce both physico-chemically different polymers under conditions of high C to N ratios. For the first time, the function and interrelation of both carbon polymers in non-diazotrophic cyanobacteria are analyzed in a comparative physiological study of single- and double-knockout mutants (Δ*glgC*; Δ*phaC*; Δ*glgC*/Δ*phaC*), respectively. Most of the observed phenotypes are explicitly related to the knockout of glycogen synthesis, highlighting the metabolic, energetic, and structural impact of this process whenever cells switch from an active, photosynthetic ‘protein status’ to a dormant ‘glycogen status’. The carbon flux regulation into glycogen granules is apparently crucial for both phycobilisome degradation and thylakoid layer disassembly in the presence of light. In contrast, PHB synthesis is definitely not involved in this primary acclimation response. Moreover, the very weak interrelations between the two carbon-polymer syntheses indicate that the regulation and role of PHB synthesis in *Synechocystis* sp. PCC 6803 is different from glycogen synthesis.

## Introduction

All microorganisms accumulate carbon biopolymers, namely glycogen and/or poly-β-hydroxybutyrate (PHB), which act as cellular sinks as well as stable and yet readily accessible reservoirs for carbon and energy, to acclimate and to cope with starvation conditions, in particular nitrogen starvation leading to high C–to-N ratios of nutrients (Allen, 1984). Glycogen is a homogeneous, water-soluble polyglucan composed of 9–13 (1–4)-linked α-D-glucose residues that are interlinked via (1–6)-α-D-glucosidic linkages, forming a highly branched and rigid granule structure of about 10^7^–10^8^ Da in mass and 42 nm in size (Ball et al., 2011). The first decisive step of glycogen biosynthesis is the synthesis of the ADP-glucose by ADP-glucose pyrophosphorylase (AGPase, GlgC; for review, see Zilliges, 2014). The glucose moiety of ADP-glucose is transferred to the non-reducing end of a linear α-(1-4) glucan chain, a reaction catalyzed by glycogen synthase (GlgA). The branching enzyme (GlgB) introduces symmetrically distributed α-(1–6) glucosidic linkages according to a binary branching principle. In contrast, PHB is a non-water-soluble, conformationally amphiphilic, linear, and highly flexible polyester consisting of (*R*)-3-hydroxybutyrate units, forming 200–500 nm large inclusions (Reusch, 2012; Jendrossek and Pfeiffer, 2014). The first step in PHB synthesis is the condensation of two molecules of acetyl-coenzyme A (CoA) to acetoacetyl-CoA, as catalyzed by β-ketothiolase (PhaA). The subsequent reduction by acetoacetyl-CoA reductase (PhaB) forms the monomeric precursor D-3-hydroxybutyryl-CoA, which is finally polymerized to PHB by PHB synthase (PhaC/PhaE; Hein et al., 1998). The PHB granule is surrounded by a membraneous surface layer containing phasin protein, which is involved in granule formation and granule attachment to cellular components (Hauf et al., 2015).

Most microorganisms synthesize either glycogen or PHB. The enzymatic settings for the metabolism of glycogen, the prevalent cyanobacterial carbon polymer, are conserved in all cyanobacteria (Beck et al., 2012). A few cyanobacteria such as *Synechocystis* sp. PCC 6803 additionally synthesize PHB (Allen, 1984; Stal, 1992; Beck et al., 2012). This dual capability is also present in some symbiotic rhizobia and some phototrophic purple bacteria, and it is an exception to the principle that only one kind of carbon-polymer metabolism acts as both a sink and an accessible reserve (De Philippis et al., 1992). In recent years, a deeper insight into the physiological role of the two physico-chemically different carbon polymers has been gained by mutagenesis approaches targeting ADP-glucose pyrophosphorylase (GlgC) and/or glycogen synthase (GlgA, for the block of glycogen synthesis; for overview, see Zilliges, 2014), and targeting ketothiolase (PhaA), and/or PHB synthase (PhaE/C), or associated granule-formation polypeptides (for the block of PHB synthesis; Taroncher-Oldenburg and Stephanopoulos, 2000; Tsang et al., 2013; van der Woude et al., 2014; Hauf et al., 2015). A variety of metabolic principles in cyanobacteria are linked to glycogen metabolism: the maintenance of photosynthetic efficiency in light and of viability in periods of starvation, such as in darkness and/or macronutrient depletion, and the acclimation to macronutrient deficiency (for overview, see Zilliges, 2014). In contrast, the role of PHB synthesis is still unclear due to the lack of a significant phenotype. The primary focus of the current study is the direct comparison of the physiological and metabolic properties of single-knockout mutants (Δ*glgC*; Δ*phaC*) with a very sensitive double-knockout mutant (Δ*glgC*/Δ*phaC*, generated in a phototrophic model organism for the first time), followed by a conclusive discussion of the individual and mutual function of both glycogen and PHB in carbon-flux regulation and in acclimation to macronutrient stress, especially during nitrogen chlorosis.

## Materials and Methods

### Mutagenesis

#### ΔphaC Mutant

For deletion–insertion mutagenesis, two equipartial, distant sequence stretches of the *phaC* ORF (locus *slr1830*) were fused by an overlap-extension PCR (primer sequences are shown in Supplementary Table S1) and ligated into the pIC20H vector (Marsh et al., 1984). A kanamycin resistance cassette from pUC4K (GE Healthcare) was finally inserted via the *Bam*HI site. The constructs were used to transform *Synechocystis* wild type as described by Ermakova et al. (1993). Transformants were restreaked six times with successively increasing antibiotic pressure. Segregation status was checked and confirmed by PCR (Supplementary Figure S1; Table S1).

#### ΔglgC Mutant

The deletion–insertion mutagenesis of the *glgC* ORF (locus *slr1176*) is described by Gruendel et al. (2012).

#### ΔglgC::glgC Complementation

For complementation of the *glgC* knockout, the *glgC* ORF (locus *slr1176*) was amplified in full length (including the promoter-region) by PCR (Supplementary Table S1). The PCR product was integrated via *Sal*I and *Pst*I into the self-replicating plasmid pVZ325. The plasmid DNA was transferred into *Synechocystis* wild-type cells and Δ*glgC*-mutant cells by triparental mating as described by Wilde and Dienst (2011). Exconjugants were restreaked six times with successively increasing antibiotic pressure, analyzed and confirmed by PCR (Supplementary Figure S2).

#### ΔglgC/ΔphaC Mutant

For double-knockout mutagenesis of both glycogen and PHB synthesis, the Δ*glgC*-knockout plasmid (Gruendel et al., 2012) was used for transformation of the Δ*phaC*-mutant cells. Transformants were restreaked six times with successively increasing antibiotic pressure, analyzed and confirmed by PCR (see Supplementary Figure S1).

### Physiological Analysis

#### Bacterial Strains and Growth Conditions

All comparative growth experiments were strictly performed without antibiotic pressure. Liquid cultures of *Synechocystis* wild-type and mutant strains were grown at 28°C under continuous illumination with white light in a range of 45–120 μmol photons m^-2^ s^-1^ in BG11 medium containing 20 mM HEPES buffer (pH 8.0) and 17.6 mM sodium nitrate as the nitrogen source (+N) or in BG11_0_, which lacks sodium nitrate (–N; Stanier et al., 1971). The growth experiments were implemented in Erlenmeyer flasks on a rotary shaker or were performed in elongated, slim flasks, which were continuously aerated in a range of 0.03-3% v/v CO_2_. Nitrogen deprivation was achieved by a repeated washing/centrifugation procedure and resuspension in BG11_0_ medium as described by Gruendel et al. (2012). The cell suspensions obtained were split, and nitrate was added to the control cell culture. Additionally, 10 mM sodium acetate (with a final concentration of 0.4% w/v) was added to the cultures in order to induce a significant PHB accumulation (after 7 days) as described by Hein et al. (1998).

Growth characteristics of the strains (in liquid cultures) have been recorded by both daily measurement of the optical density at 750 nm (OD_750_) and of the chlorophyll a content (determination according to the method as described by Tandeau de Marsac and Houmard, 1988), recorded by Specord200 plus spectrophotometer (Analytic Jena AG, Germany) (Supplementary Figure S3).

To monitor the bleaching process (under nitrogen deprivation), whole-cell absorption spectra have been recorded in the wavelength range from 400 to 750 nm on a Specord200 plus spectrophotometer (Analytic Jena AG, Germany) equipped with a scattering position and were normalized at 750 nm (Supplementary Figure S4).

#### Viability Tests (Spot Assay Experiments)

**FIGURE 1:**
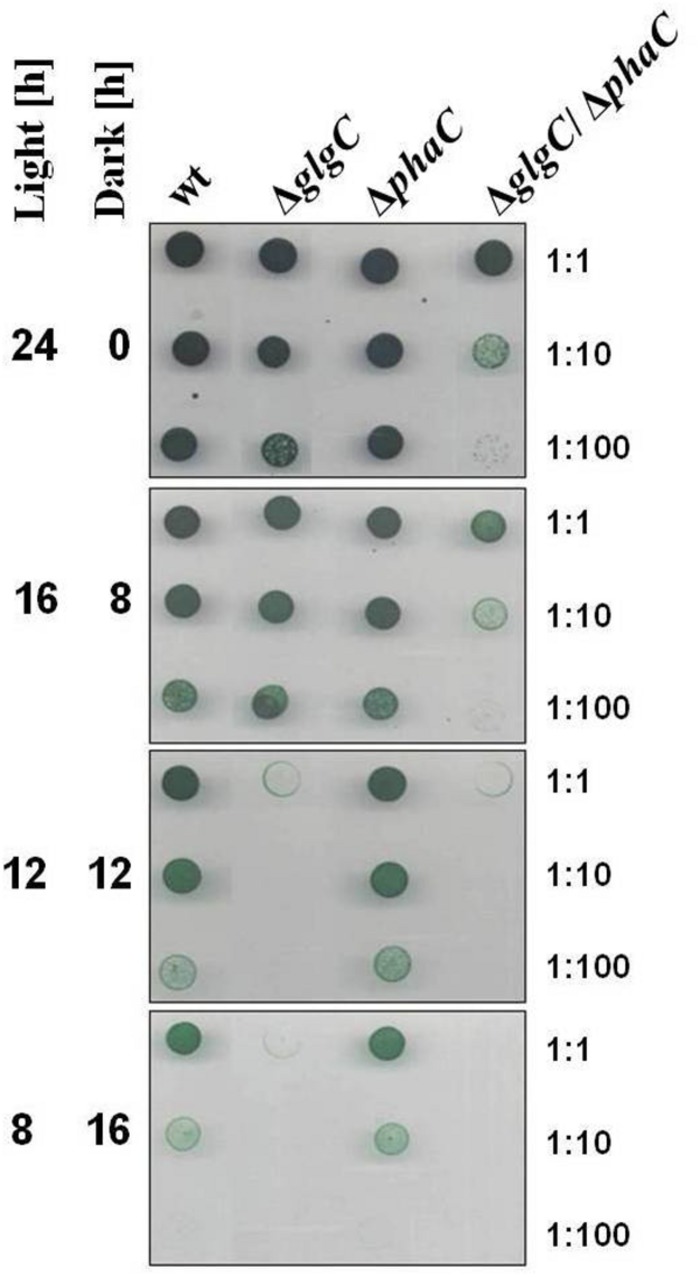
**Viability test under alternating light-dark conditions.** Growth and viability of the wild type (wt) and mutants defective in glycogen synthesis (Δ*glgC*), PHB synthesis (Δ*phaC*), or both carbon-polymer syntheses (Δ*glgC*/Δ*phaC*) were tested via spot assays (7 days) under continuous and alternating light/dark (16 h/8 h, 12 h/12 h, or 8 h/16h) conditions (see Materials and Methods). The effect was verified by three independent spot assays.

**FIGURE 2:**
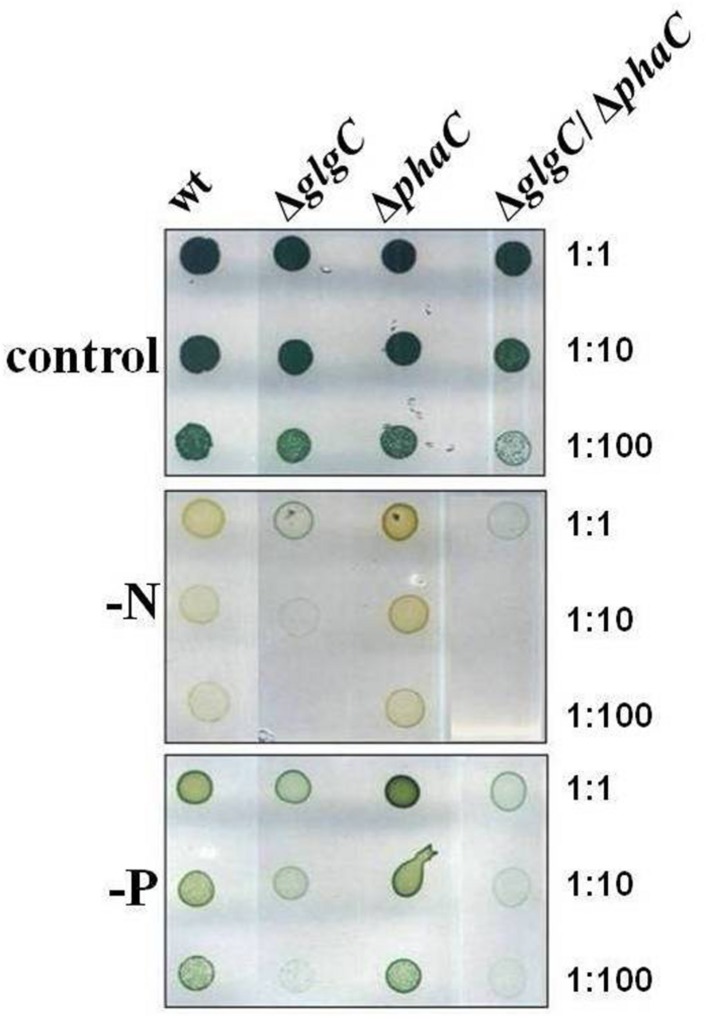
**Viability test under nitrogen and phosphate depletion.** Growth and viability of the wild type (wt) and mutants defective in glycogen synthesis (Δ*glgC*), PHB synthesis (Δ*phaC*), or both carbon-polymer syntheses (Δ*glgC*/Δ*phaC*) were tested via spot assays under continuous illumination (7 days) on BG11 agar plates lacking the respective nitrogen (–N) or phosphate (–P) source (see Materials and Methods). The effect was verified by four independent spot assays.

**FIGURE 3:**
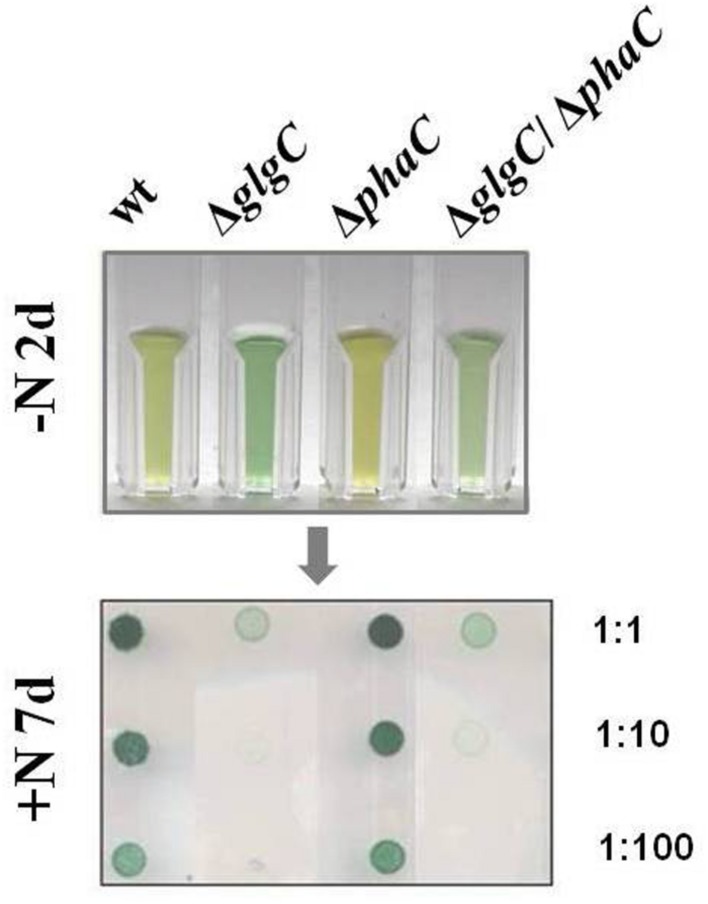
**Recovery capability.** The recovery capability of nitrogen-depleted (–N, 2 days) wild type (wt) and mutants defective in glycogen synthesis (Δ*glgC*), PHB synthesis (Δ*phaC*), or both carbon-polymer syntheses (Δ*glgC*/Δ*phaC*) were tested via spot assays under continuous illumination on BG11 agar plates replenished with the nitrogen source (+N; see Materials and Methods). The effect was verified by six independent spot assays.

For spot assay experiments, cells were grown in Erlenmeyer flasks to an OD_750nm_ of 0.4–0.6 and adjusted to a chlorophyll content of 5 μg ml^-1^ (chlorophyll determination according to the method as described by Tandeau de Marsac and Houmard (1988). Seven microliters aliquots of three different dilutions (1:1, 1:10, 1:100) were dropped on BG11 agar plates with (+N) or without NaNO_3_ (–N) and with or without K_2_HPO_4_ (+P_i_ or –P_i_), respectively. The plates were incubated at 28°C under alternating light/dark cycles or continuous illumination with a light intensity of 75 μmol photons m^-2^ s^-1^ for 7 days (**Figures 1–3**).

### Determination of Metabolite Concentrations

#### Quantitative Determination of Glycogen (Glc_n_)

**FIGURE 4:**
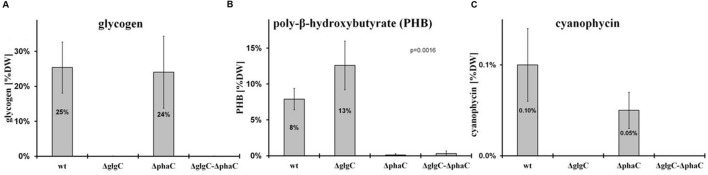
**Polymer-accumulation profile analysis.** The accumulation of polymers in wild type (wt) and mutants defective in glycogen synthesis (Δ*glgC*), PHB synthesis (Δ*phaC*), or both carbon-polymer syntheses (Δ*glgC*/Δ*phaC*) were recorded for glycogen after 2 days of nitrogen depletion **(A)**, for PHB after 7 days of nitrogen depletion **(B)**, and for cyanophycin after 24 h of replenished nitrogen conditions [following 2 days of nitrogen depletion **(C)** (see MATERIALS AND METHODS)]. Data from glycogen accumulation **(A)** represent mean values and standard deviations from 11 different growth experiments, data from PHB **(B)** and cyanophycin **(C)** accumulation represent mean values and standard deviations from 3 to 5 different experiments (with three biological replicates each). The *p*-value (significance level) was calculated by Welch’s *t*-test. Metabolite levels are calculated (monomers per dry weight in mg/g) as follows: **(A)** glycogen from glucose equivalents, **(B)** PHB from β-hydroxybutyrate monomers, and **(C)** cyanophycin from β-Aspartylarginine dipeptides related to the relative amounts of cell dry weight (%DW).

The intracellular glycogen levels were determined according to the protocol as described by Gruendel et al. (2012) (**Figure 4A**).

#### Quantitative Determination of PHB

Poly-β-hydroxybutyrate was extracted from dried cell pellets and simultaneously hydrolyzed into its monomers (3-hydroxybutyrate) by shaking in 0.5 M NaOH (300–400 μl) (1 h incubation at 85°C). After cooling, the solution was neutralized by the addition of 1 M HCl (volume ratio NaOH/HCl 4:1). Finally, the PHB content was spectrophotometrically quantified in an enzymatic assay using 3-hydroxybutyrate dehydrogenase from *Rhodobacter spheroides* (Roche Diagnostics) in the presence of nicotinamide adenosine dinucleotide (NAD). The NADH synthesis was coupled to a phenazine methosulphate-p-iodonitrotetrazolium violet (PMS-INT) colorimetric assay (to prevent a backward reaction) as described by Lim and Buttery (1977). Here, the generated NADH transfers its hydrogen through the PMS-INT system to produce a red formazan, which is stable and absorbs light at 505 nm. The PHB quantification (**Figure 4B**) was performed via the calibration of NADH series.

#### Quantitative Determination of Cyanophycin (CyPh) Content

Cyanophycin was extracted as described by Li et al. (2001) using a french press instead of a sonifier. The cyanophycin pellet was hydrolyzed as described by Maheswaran et al. (2006) and quantified enzymatically as described by Bergmeyer et al. (1974) (**Figure 4C**).

#### Quantitative Determination of Extracellular Pyruvate and 2-Oxoglutarate

The extracellular pyruvate and 2-oxoglutarate levels were determined according to the protocol as described by Gruendel et al. (2012) (**Table 1**).

**Table 1:** Exclusive spilling of 2-oxoglutaric acid and pyruvic acid in glycogen-deficient mutants.

	wt	Δ*phaC*	Δ*glgC*	Δ*glgC:: glgC*	Δ*glgC*/ Δ*phaC*
2-OG	n.d.	n.d.	0.1–0.2	n.d.	0.2–0.3
Pyr	n.d.	n.d.	0.3–0.6	n.d.	0.3–0.8

*The extracellular levels of 2-oxoglutaric acid (2-OG) and pyruvic acid (Pyr; in mM concentrations; n.d. not detectable) were determined in the supernatant of the wild type (wt), of mutants defective in glycogen synthesis (ΔglgC), PHB synthesis (ΔphaC), or both carbon-polymer syntheses (ΔglgC/ΔphaC), as well as of the ΔglgC mutant, complemented with the glgC gene in trans (ΔglgC::glgC), after 48 h of nitrogen depletion (see MATERIALS AND METHODS). Data from determination of extracellular pyruvate and 2-oxoglutarate represent variances from 6 different growth experiments á 3 biological replicates.*

#### Quantitative Determination of Dry Weight

For the determination of dry weight, cell pellets of 10–50 ml culture were washed twice with sterile water, dried completely at 80°C in a heating device, and finally weighed.

### Transmission Electron Microscopy

**FIGURE 5:**
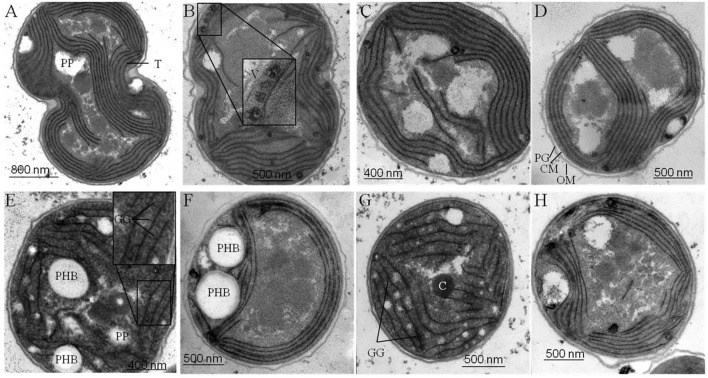
**Ultra-structural changes in response to nitrogen starvation.** Structural changes occurring in wild type (wt) **(A, E)** and mutant strains defective in glycogen synthesis (Δ*glgC*) **(B, F)**, in PHB synthesis (Δ*phaC*) **(C, G)**, or in both carbon-polymer syntheses (Δ*glgC*/Δ*phaC*) **(D, H)** in response to nitrogen deprivation were monitored by transmission electron microscopy. Cells deprived of nitrogen (2 days) **(E–H)** are compared with respective, untreated controls **(A–D)** (see Materials and Methods). T, thylakoids; GG, glycogen granule; PHB, polyhydroxybutyrate granule; C, carboxysome; PP, polyphosphate granule; V, vesicle; PG, peptidoglycan layer; CM, cytoplasmic membrane; OM, outer membrane.

Samples for Transmission electron microscopy (TEM) were prepared as described previously for *Anabaena* sp. PCC 7120 (Fiedler et al., 1998). Fixation and post-fixation of the cells were performed using 2.5% (v/v) glutaraldehyde and 2% (w/v) potassium permanganate. Fixed cells were immobilized in agarose and dehydrated by a series of increasing ethanol. After embedding in EPON and sectioning, the ultrathin sections (60–80 nm) were stained with uranyl acetate and lead citrate. The sections were examined with a Philipps Tecnai electron microscope at 80 kV (**Figure 5**).

## Results

### Double Mutants Lacking Glycogen and PHB

To completely abolish carbon-polymer biosynthesis, knockout mutants of either ADP-glucose pyrophosphorylase (AGPase, GlgC) or glycogen synthase (GlgA) for glycogen biosynthesis, or of either β-ketothiolase (PhaA), Acetoacetyl-CoA reductase (PhaB), or PHB synthase (PhaC/PhaE) for PHB synthesis were successfully created in different cyanobacterial strains previously (Taroncher-Oldenburg and Stephanopoulos, 2000; Xie et al., 2011; Tsang et al., 2013; van der Woude et al., 2014; Zilliges, 2014). In this study, we describe for the first time the generation of a double-knockout mutant lacking both AGPase (GlgC) and PHB synthase (PhaC) (see Supplementary Figure S1). The resulting strain (Δ*glgC*/Δ*phaC*) is not able to synthesize neither glycogen nor PHB (see below). The direct comparison of this mutant with the respective single-knockout mutant and the wild type (as well as a complemented variant, see Supplementary Figure S2 for *glgC*) provide a deeper understanding of the individual and the mutual physiological roles as well as the metabolic interrelations of the two carbon-polymer syntheses.

### Energy and Carbon Sources for the Maintenance Metabolism in Darkness

All cyanobacteria are able to perform a maintenance metabolism in the absence of light by predominantly using internal polymer reserves. Various studies showed the contribution of the carbon storage polymer glycogen to the maintenance metabolism in darkness. In contrast, the involvement of PHB metabolism has not been reported so far (for review, see Zilliges, 2014). In order to directly compare and demonstrate putative interrelations between the two carbon polymer syntheses, the growth and viability of a glycogen-deficient mutant (Δ*glgC*), of a PHB-deficient mutant (Δ*phaC*), and of a mutant deficient in the synthesis of both compounds (Δ*glgC*/Δ*phaC*) have been tested via spot assays as well as in liquid culture systems under different alternating light/dark regimes (**Figure 1** and Supplementary Figures S3A,B). The Δ*phaC* mutant shows similar viability and growth as the wild type under all tested conditions (**Figure 1**; Supplementary Figures S3A,B). In contrast, the lack of glycogen in the Δ*glgC* strain has a negative effect on viability and growth with increasing length of periodic darkness (**Figure 1**; Supplementary Figures S3A,B). Only under continuous low-light regimes, the viability and growth of the Δ*glgC* mutant and the wild type are similar (**Figure 1** and Supplementary Figures S3A,B). In contrast, the double-knockout mutant Δ*glgC*/Δ*phaC* shows reduced viability even under continuous low-light conditions (**Figure 1**). Moreover, the negative effect on the viability and growth with increasing length of periodic darkness is more pronounced in the Δ*glgC*/Δ*phaC* mutant than in the Δ*glgC* single mutant. Cells deficient in both PHB and glycogen synthesis are not able to grow in conditions of periodic 12 h/12 h light/dark regimes (**Figure 1**). In conclusion, both carbon-polymer syntheses are presumably essential for the photoautotrophic lifestyle of *Synechocystis* sp. PCC 6803. However, the glycogen pool (and not the PHB pool) might be the main energy and carbon source for the maintenance metabolism in darkness and the PHB pool is dispensable.

### Carbon-Polymer Synthesis as an Adaptation and Survival Strategy to Macronutrient Stress Conditions

Unbalanced growth conditions such as macronutrient starvation generally lead to an accumulation of reserve material as well as to a rapid degradation after replenishing conditions in all microorganisms including cyanobacteria (Schwarz and Forchhammer, 2005). To understand the role and interrelations between the two carbon polymer metabolisms in such stress responses, the growth and viability of the double-knockout mutant Δ*glgC*/Δ*phaC* in comparison to both single-knockout mutants Δ*glgC* and Δ*phaC* have been tested in response to nitrogen and phosphate depletion (**Figure 2** and Supplementary Figure S3C) as well as after nitrogen-replenishing conditions (**Figure 3**). The Δ*glgC* mutant is significantly impaired in both its viability (**Figure 2**) and its recovery capability (**Figure 3**) in prolonged starvation condition, as previously reported by Gruendel et al. (2012).

The single, PHB-deficient mutant Δ*phaC* behaves similar to the wild type. Upon nitrogen starvation both strains duplicate once more and enter chlorosis, being visualized by the yellow color due to the loss of pigments (**Figure 2**; Supplementary Figure S3C). Upon Pi depletion, the strains show reduced growth and only faint yellow-green pigmentation (**Figure 2**). The Δ*phaC* mutant recovers after starvation stress like the wild type, tested after 7 days of nitrogen depletion (**Figure 3**).

In line with this finding, the Δ*glgC*/Δ*phaC* mutant shows a similar phenotype as the Δ*glgC* mutant, indicating that the impaired growth, viability, and recovery capacity result rather from the block of glycogen synthesis than from the block of PHB synthesis in this double-knockout mutant (**Figures 2** and **3**). In conclusion, the glycogen synthesis (and not the PHB synthesis) appears to be the key player in *Synechocystis*’s short-term acclimation response toward macronutrient stress conditions, such as nitrogen starvation.

### Metabolic Consequences of Blocked Carbon-Polymer Synthesis upon Macronutrient-Stress Response

As previously shown, the block of glycogen biosynthesis as in the Δ*glgC-* or Δ*glgA_n_*-mutant strains leads to three very characteristic, co-occurring phenotypes as short-term response to nitrogen starvation: (i) the incapability of carbon-excess redirection into glycogen-granule formation (see also **Figures 4** and **5**), (ii) impaired chlorosis (non-bleaching phenotype; (**Figures 2**, **3**; Supplementary Figure S4), and (iii) the spilling of partially oxidized carbonic acids, such as 2-oxoglutarate and pyruvate (**Table 1**; for overview, see Zilliges, 2014). All three characteristics are also observed with a similar magnitude under the tested conditions in the double-knockout mutant Δ*glgC*/Δ*phaC* (**Figures 2**–**5**; Supplementary Figure S4; **Table 1**). This phenotype is fully complemented by *in-trans* expression of the *glgC* gene under the control of its own promoter in the Δ*glgC*-mutant background (data shown for overflow metabolism only; see **Table 1**: Δ*glgC*::*glgC*) and is in agreement with results from Carrieri et al. (2012) using a similar complementation approach. In contrast, the Δ*phaC* mutant responds to nitrogen-starvation conditions in a similar way as the wild type, which is in agreement with previous data obtained with a Δ*phaA* mutant (van der Woude et al., 2014). Both wild-type and Δ*phaC* mutant accumulate similar amounts of glycogen (approx. 25% of DW in 48 h) (**Figure 4**), degrade their phycobilisomes in a similar period (approx. 48 h; Supplementary Figure S4), and do not spill any of the partially oxidized carbonic acids such as 2-oxoglutarate (**Table 1**). Under the nitrogen-replenishing conditions tested, the wild type and the Δ*phaC* mutant show a similar glycogen-degradation pattern (data not shown) and a similar cyanophycin accumulation (**Figure 4**).

In contrast, the glycogen-deficient mutants Δ*glgC* and Δ*glgC*/Δ*phaC* are apparently not able to redirect the replenished nitrogen sources into cyanophycin biosynthesis, which is not detectable in cell extracts under the tested conditions (**Figure 4**). However, in response to nitrogen-starvation conditions, the single-knockout mutant Δ*glgC* significantly (**Figure 4**: *p*-value below 0.01) redirect more of the carbon excess into PHB biosynthesis than the respective wild type, which is in agreement with data from Wu et al. (2001).

In conclusion, blocks in glycogen synthesis (but not blocks in PHB synthesis) lead to overflow reactions into other polymer syntheses (and apparently not *vice versa*) as well as to a spilling of certain metabolites such as 2-oxoglutarate in response to macronutrient starvation.

### Effect on Cellular Ultra-Structure by Lack of Carbon-Polymer Biosyntheses

Four major structural and morphological changes are observed in wild-type cells in response to nitrogen starvation (**Figure 5**): (i) a massive accumulation of electron-dense glycogen inclusions (approx. 40 nm in diameter) between the thylakoid layers (**Figure 5E**), (ii) the degradation of the antenna complexes (phycobilisomes; Supplemantary Figure S4), (iii) the disassembling of the thylakoid membrane layers including a reduction by number and packing density (**Figure 5E**), and (iv) the formation of distinct electron-transparent PHB granules (approx. 400–500 nm in diameter; **Figure 5E**). Similar structural and morphological changes, except for the accumulation of PHB inclusions, occur in the PHB synthase-deficient mutant Δ*phaC* (**Figures 5C,G** and Supplementary Figure S4). This finding is in agreement with previously published data by Tsang et al. (2013). Some of the electron-transparent structures in both cell types are presumably polyphosphate granules (indicated in **Figure 5A**) as previously highlighted by Tsang et al. (2013) and Nagarajan et al. (2014).

In contrast, AGPase-deficient mutant cells (Δ*glgC*; **Figures 5B,F**; Supplementary Figure S4) as well as glycogen synthase-deficient cells (Δ*glgA1/*Δ*glgA2*; data not shown) are not able neither to degrade their antenna complexes (phycobilisomes), nor to reduce the number and packing density of the thylakoid membrane-layer system, nor to accumulate glycogen granules in response to nitrogen depletion. Instead, several undefined vesicles (approx. 90 nm in diameter) appear inside the thylakoid stacks (**Figures 5B,F**). Preparative artifacts could be excluded due to equal treatment of all samples (**Figure 5**; compare to wild type and Δ*phaC* mutant). A similar effect of structural and morphological changes as in Δ*glgC*-mutant cells, except for the formation of definitive PHB granules, has been observed in the double-knockout mutant Δ*glgC/*Δ*phaC* (**Figures 5D,H**).

In conclusion, the glycogen synthesis (and not the PHB synthesis) has a substantial influence on the adaptation response to macronutrient starvation. The formation of glycogen granules appears as an important key player in the rearrangement and disassembling of the photosynthetic machinery such as phycobilisome degradation and thylakoid density and packing alterations. These changes are necessary to acclimate and trophically convert from an active photosynthetic cell to a dormant status.

## Discussion

### Glycogen Synthesis as a Metabolic and Structural Key Process in Nitrogen Acclimation

**FIGURE 6:**
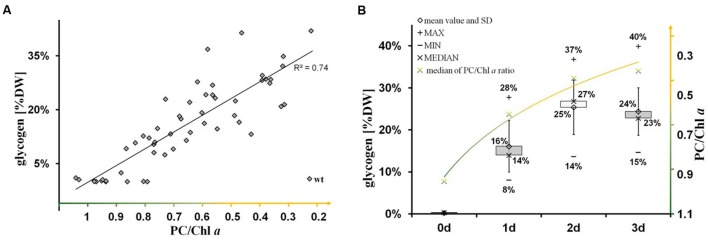
**Significant linear correlation between the progression of glycogen synthesis and nitrogen chlorosis. (A)** The significance of linear correlation (indicated by the coefficient of determination *R*^2^ is 0.74) between the two groups (glycogen accumulation and progression of nitrogen chlorosis), as shown in the scatter plot, has been determined by a *t*-test using EXCEL TDIST (two-tailed) program. Hereby, the *p*-value has been calculated to 1.52 × 10^-25^ regarding the test statistic of 12.2 and the degrees of freedom of *f* = 54-2. **(B)** Time-dependent course (0–3 days) of glycogen accumulation (left axis, gray black boxes including data labels in % (w/w) glycogen/dry weight) and phycobilisome degradation (right axis, green-yellow labels in ratios of phycocyanin to chlorophyll *a*) after the onset to nitrogen deprivation in wild-type cells. A similar time-course was observed for Δ*phaC*-mutant cells (data not shown). The progression of phycobilisome degradation (phycocyanin peak at 628 nm) was calculated in relation to the chlorophyll *a* peak (PC/Chl) by analysis of whole-cell absorption spectra. Each time point is represented by more than 10 single pair values indicating the range (minimum and maximum) and the average (median and arithmetic mean).

The nitrogen-starvation response is characterized by a cascade of structural and metabolic events that finally switch the cellular mode from photosynthetically active to dormant status in non-diazotrophic cyanobacteria. These substantial changes are tightly related to active glycogen synthesis. The removal of external nitrate or ammonia sources stops the primary nitrogen assimilation processes mainly mediated by glutamine synthetase and glutamine/2-oxoglutarate aminotransferase. The reduction of cellular nitrogen is accompanied by the rapid rise of 2-oxoglutarate (2-OG) levels due to the absence of 2-OG dehydrogenase (2-OGDH; Muro-Pastor et al., 2001). Even in 2-OGDH-containing bacteria and plants, the enzyme is almost always inhibited during nitrogen starvation, which leads in turn to a concomitant overflow of the carbon excess into either glycogen or PHB polymer synthesis, amino acid biosynthesis, or alternative TCA shunts (e.g., GABA shunt; Walshaw et al., 1997). Our comparative study of knockout mutants reveals unambiguously that the primary carbon excess (from terminating photosynthetic reactions and starting amino acid release by protein degradation (Hasunuma et al., 2013; Depraetere et al., 2015) is mainly directed into glycogen synthesis and not into PHB synthesis (**Figure 4**). Only blocks of glycogen synthesis in *Synechocystis* sp. PCC 6803 lead to a prompt spilling of partially oxidized substrates such as 2-OG (to get rid of carbon excess) under unbalanced growth conditions, independent of the absence or presence of an active PHB synthesis (**Table 1**). However, this spilling effect is similar to PHB-synthesis knockouts in other bacteria naturally lacking glycogen metabolism (Steinbuchel and Schlegel, 1989; Russell, 2007). The directing of carbon excess into glycogen synthesis (and not into PHB synthesis) is crucial for an adequate acclimation from an active photosynthetic cell to a dormant cell in the presence of light. In the absence of external nitrogen sources, both light-dependent processes, the progression of chlorosis (Gorl et al., 1998) and the glycogen synthesis (Gruendel et al., 2012), are inextricably linked with each other (**Figure 6**; Supplementary Figure S4). Statistical data analysis indicates a significant linear correlation (coefficient of determination *R*^2^ is 0.74; Pearson coefficient is 0.86; *p*-value is 1.52 × 10^-25^) between the progression of both glycogen accumulation and phycobilisome degradation in wild-type cells (**Figure 6**; Supplementary Figure S4) and in Δ*phaC* cells (data not shown) as a short-term response to nitrogen depletion. Cells deficient in glycogen synthesis are not able to perform the switch and remain in a metabolic arrest (**Figures 5B,F**).

We hypothesize two mechanisms that do not necessarily exclude each other: a metabolic mechanism (i) and/or a structural mechanism (ii). (i) The concomitant directing of carbon excess (mainly from protein degradation) into glycogen synthesis allows the adjustment to an adequate cellular 2-OG concentration under conditions of nitrogen depletion, which in turn leads to an activation of the nitrogen signaling cascade, in particular of the PII signaling protein (Muro-Pastor et al., 2001; Schwarz and Forchhammer, 2005). An insufficient activation of enzymes and associated proteins of the nitrogen signaling cascade most likely prevents nitrogen chlorosis, similar as shown for *nblA* in *Synechococcus elongatus* PCC 7942 by Hickman et al. (2013). (ii) The replacement of phycobilisomes (around 35–40 nm in size) by rigid glycogen granules (around 42 nm in size) between the thylakoid layers is a crucial structural component for the subsequent, controlled shut-down process of photosynthetic activity including thylakoid disassembly. Likewise, the AGPase-deficient mutant (Δ*glgC*) of *Synechococcus* sp. PCC 7002 still maintained an efficient energy transfer from phycobilisomes to photosystem II reaction centers after prolonged nitrogen starvation (Jackson et al., 2015). The increased abundance of yet undefined vesicles between the thylakoid layers in the Δ*glgC* mutant of *Synechocystis* sp. PCC 6803 (**Figure 5B**) additionally supports the new role of glycogen synthesis/granules for the integrity of the thylakoid layer system. Such vesicles (around 90 nm in size) might represent spilling products (to remove destabilizing catabolites from the thylakoid membrane) or plastoglobuli-like structures, which have similar functions in the thylakoid biogenesis/degradation processes in plants (Brehelin and Kessler, 2008; Cunningham et al., 2010; Besagni and Kessler, 2013). A potential confusion with lipid droplets is not likely due to size and shape of the vesicles (Peramuna and Summers, 2014; Perez et al., 2015).

### The Classical Storage Role and Potential Interrelations of Both Carbon Polymers

This comparative study shows that the glycogen and PHB biosyntheses do not negatively affect each other (**Figure 4**) and thus potentially do not interrelate under conditions of nitrogen starvation. Only a small proportion of primary carbon excess is redirected into PHB synthesis in cells blocked in glycogen synthesis (**Figure 4**). Most excessive carbon proportions are promptly spilled (**Table 1**). In this respect, it is not clarified whether parts of the carbon excess were directed into lipid biosynthesis, another abundant form of carbon storage in *Synechocystis* sp. PCC 6803 (Monshupanee and Incharoensakdi, 2014). Lipid droplets as observed in akinetes of *Nostocales* were not detected in electron micrographs of wild type and mutant cells (**Figure 5**; compare Perez et al., 2015). Moreover, this and several other physiological studies show that massive glycogen synthesis starts promptly with the onset of nitrogen starvation. Significant amounts of glycogen are already detectable after one day, peaking around 3 days of nitrogen starvation, whereas PHB does not peak until after five days of the nitrogen-starvation conditions tested (Lehmann and Wober, 1976; De Philippis et al., 1992; Miyake et al., 1997; Panda et al., 2006; Monshupanee and Incharoensakdi, 2014; Hauf et al., 2015). This temporal shift in the nitrogen response of the two carbon-polymer syntheses is in agreement with transcriptional data (Krasikov et al., 2012; Huang et al., 2013; Kopf et al., 2014; Depraetere et al., 2015). A concomitant expression has only been shown for enzymes involved in glycogen degradation, such as the glycogen phosphorylase (GlgP2) and debranching enzyme (GlgX), as well as for PHB synthesis enzymes, such as ketothiolase (PhaA), acetyl-coenzyme A reductase (PhaB), and PHB synthase (PhaCE; Azuma et al., 2011; Osanai et al., 2013; Nakaya et al., 2015). Both catabolic enzymes (GlgP2, GlgX) are potentially also involved in glycogen modification, which changes the accessibility of the polymer for short- or long-term storage (Suzuki et al., 2007). Finally, we cannot conclusively answer whether the carbon polymers glycogen and PHB serve as storage compounds for *Synechocystis*’ maintenance metabolism during macronutrient-starvation periods.

The recovery incapability of the glycogen-deficient mutants (**Figure 3**) possibly results from the variety of negative pleiotropic (metabolic and structural) effects, summarized in an impaired acclimation response, than from the lack of carbon source. Solely PHB-deficient cells are neither affected under the conditions tested (**Figures 1–3**), which is in agreement with Tsang et al. (2013), van der Woude et al. (2014), and Hauf et al. (2015). A longer starvation exposure (above seven days), the determination of polymer modifications under nitrogen starvation and replenished conditions will most certainly bring new indications regarding the physiological function. Moreover, it cannot be conclusively answered why a few cyanobacterial species such as *Synechocystis* sp. PCC 6803 (and some purple bacteria) massively synthesize both types of the physically and chemically different carbon polymers, glycogen, and PHB, in response to macronutrient deficiency (**Figure 4**). Obviously, PHB synthesis does not act as a backup of glycogen synthesis and does not compensate knockouts in glycogen metabolism.

These observations are in line with the general metabolic principle that only one kind of carbon-polymer metabolism acts as both a sink and an accessible reserve. So far, the only indication for the specific role of PHB in *Synechocystis* sp. PCC 6803 is given by the pronounced sensitivity and reduced growth rate of the double-knockout mutant Δ*glgC*/Δ*phaC* (certain loss of robustness) compared to the single knockouts even under standard growth conditions (**Figure 1**). In this respect, some already discussed functions of PHB are conceivable: (i) as a transient electron sink (Stal, 1992) and/or (ii) as storage component (however questioned by the lack of specific enzymes for PHB degradation in *Synechocystis* sp. PCC 6803 and the non-compensatory effect in glycogen-deficient knockouts), and/or (iii) as a structural component during the equal separation of the multiple bacterial nucleoids and PHB granules in cell division processes (Jendrossek and Pfeiffer, 2014), that might be essential for an immediate re-start of cell proliferation after replenished nitrogen conditions.

## Author Contributions

RD, IM, and YZ made substantial contributions to the conception and the design of the study, the acquisition of data, and the analysis and interpretation of data; RD, IM, and YZ participated in drafting the article and revising it critically for important intellectual content; RD, IM, and YZ gave final approval of the version to be submitted. RD, IM, and YZ agreed to be accountable for all aspects of the work in ensuring that questions related to the accuracy or integrity of any part of the work are appropriately investigated and resolved.

## Conflict of Interest Statement

The authors declare that the research was conducted in the absence of any commercial or financial relationships that could be construed as a potential conflict of interest.
