# Supplementary material for: The Multiple Functions of Common Microbial Carbon Polymers, Glycogen and PHB, during Stress Responses in the Non-Diazotrophic Cyanobacterium Synechocystis sp. PCC 6803
Source: Front Microbiol. 2016 Jun 21;7:966. doi: 10.3389/fmicb.2016.00966 (PMC4914499; doi:10.3389/fmicb.2016.00966)
Supplement: Supplementary file 1 [file Data_Sheet_1.DOCX]

**SUPPLEMENT**

**Table S1: Primer list.** Listed primers for knockout mutagenesis of the *glgC* gene (glgC.fw, glgC.rv) and the *phaC* gene (phaC_XbaI.fw, phaC_BamHI.rv; phaC_BamHI.fw, phaC_ClaI.rv) for *in-trans* complementation of the *glgC* knockout (glgC_SalI.fw, glgC_PstI.rv) and for segregation analysis (**see Fig. S1 and S2**).

|  | **name** | **sequence** |
| --- | --- | --- |
| **cloning** | phaC_**XbaI**.fw | CCGATG**TCTAGA**TAATTCACCATC |
|  | phaC_**BamHI**.rv | TCTAGGG**GGATCC**AACGATCG |
|  | phaC_**BamHI**.fw | CCAGG**GGATCC**TCTTAACCTAG |
|  | phaC_**ClaI**.rv | TGTCGT**ATCGAT**AGCCAATGG |
|  | glgC.fw | ACCCCATCATCATACGAAGC |
|  | glgC.rv | GTCTGCCGGTTTGAAACAAT |
|  | glgC_**SalI**.fw | **GTCGAC**AACCTAGTCAGCTCCCCAGA |
|  | glgC_**PstI**.rv | **CTGCAG**GCTATGGTGCGAGGAAAGAA |
| **check** | phaC_KO.fw | TCCTCAAACTGGGTTTGGAC |
|  | phaC_KO.rv | GCAGGGGTTTAAGCATGAGA |
|  | glgC_KO.fw | TCCCGTCAGTAATTGCATCA |
|  | glgC_KO.rv | AGCCTCCTGGACATTTTCCT |
|  | glgC_OX.fw | TTAGCCCTGACCAAACAACC |
|  | pVZ-seq.rv | GCTTTCCTGGCTTTGCTTCC |

**
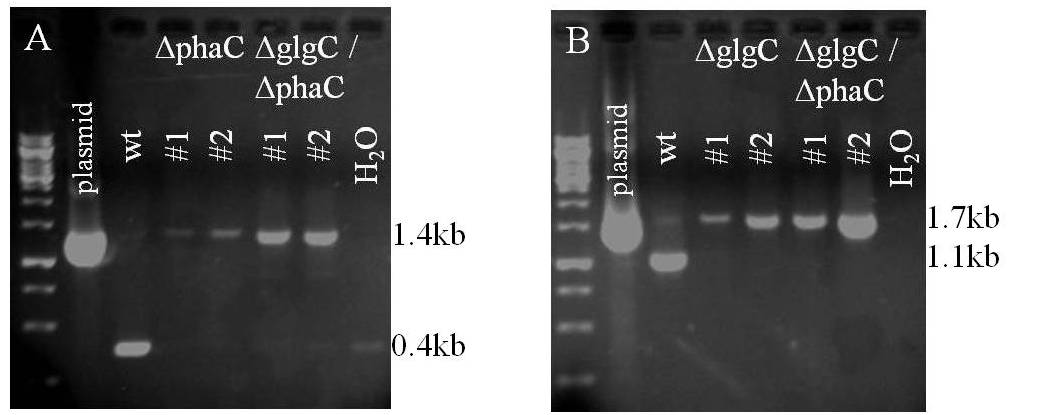
**

**Figure S1: Complete segregation of carbon-polymer knockout mutagenesis.** The segregation status of mutants defective in glycogen synthesis (Δ*glgC*), PHB synthesis (Δ*phaC*), or both carbon-polymer syntheses (Δ*glgC*/Δ*phaC*) were determined by colony-PCR. The *phaC* gene region (**A**) was amplified by phaC_KO.fw and phaC_KO.rv primers (**Tab. S1**), resulting in a 0.4 kb (genetically non-mutated) fragment or a 1.4 kb (genetically mutated) fragment, respectively; the *glgC* gene region (**B**) was amplified by glgC_KO.fw and glgC_KO.rv primers (**Tab. S1**), resulting in a 1.1 kb (genetically non-mutated) fragment or a 1.7 kb (genetically mutated) fragment. The plasmid DNA of the respective genetic knockout construct and genomic DNA of the wild type were used as controls.

**
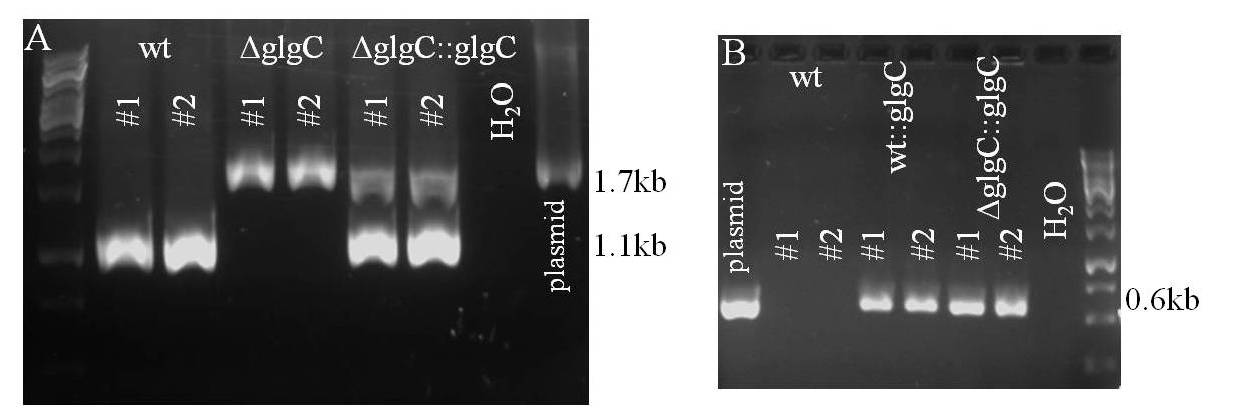
**

**Figure S2: *In-trans* complementation of *glgC* knockout in theΔ*glgC::glgC* strain.** The genomic segregation status of the *glgC* knockout (**A**) inside the revertant was checked and confirmed by colony-PCR using glgC_KO.fw and glgC_KO.rv primers (**Tab. S1**), resulting in a 1.7 kb fragment. The *glgC* gene encoded *in-trans* in the plasmid (**B**) was detected by colony-PCR using glgC_OX.fw and pVZ_seq.rv primers (**Tab. S1**), resulting in a 0.6 kb fragment. The plasmid DNA of the respective genetic knockout construct and genomic DNA of the wild type were used as controls.

**
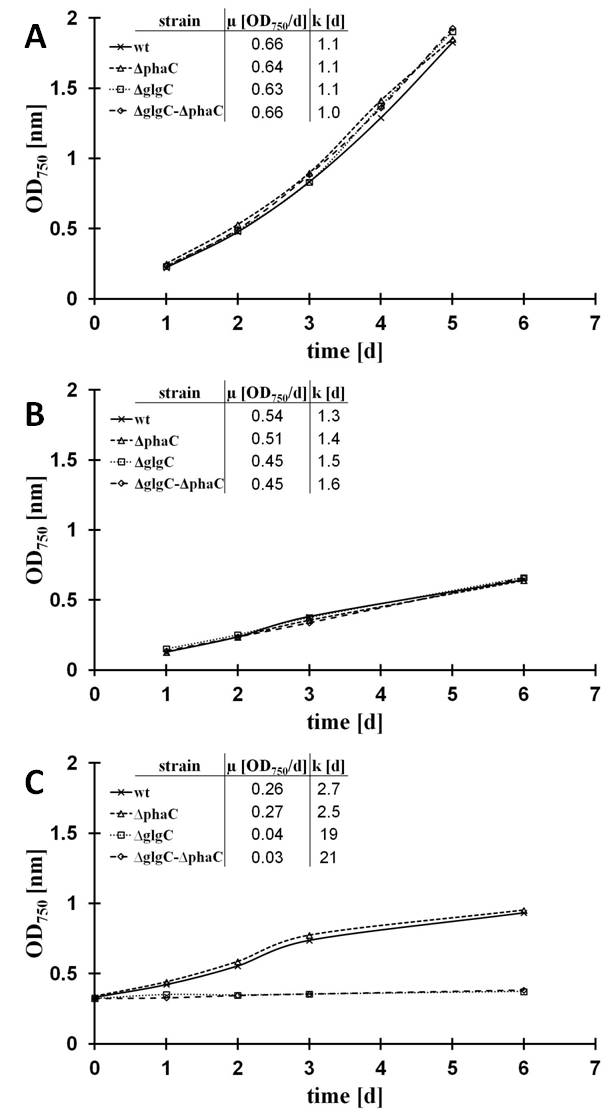
**

**Figure S3: Growth characteristics of carbon-polymer knockout mutants in liquid cultures** under continuous illumination **(A)**, light (16 hours)/ dark (8 hours) cycle conditions **(B)** and after the onset to nitrogen starvation **(C).** All cultures (wt, ΔglgC, ΔphaC, ΔglgC/ΔphaC) were grown in BG11 medium (or BG11_0_ medium, **(C)**), illuminated with white light of 45 µE m^-2^ s^-1^ (except for dark period, **(B)**) and aerated with 0.05% (v/v) CO_2_. The growth was monitored by both daily measurements of optical density at 750nm and of chlorophyll a content. The growth rates (µ in OD_750_ increment per day) and corresponding doubling times (k in days) were calculated as a linear regression of the natural logarithm of the optical density.

**
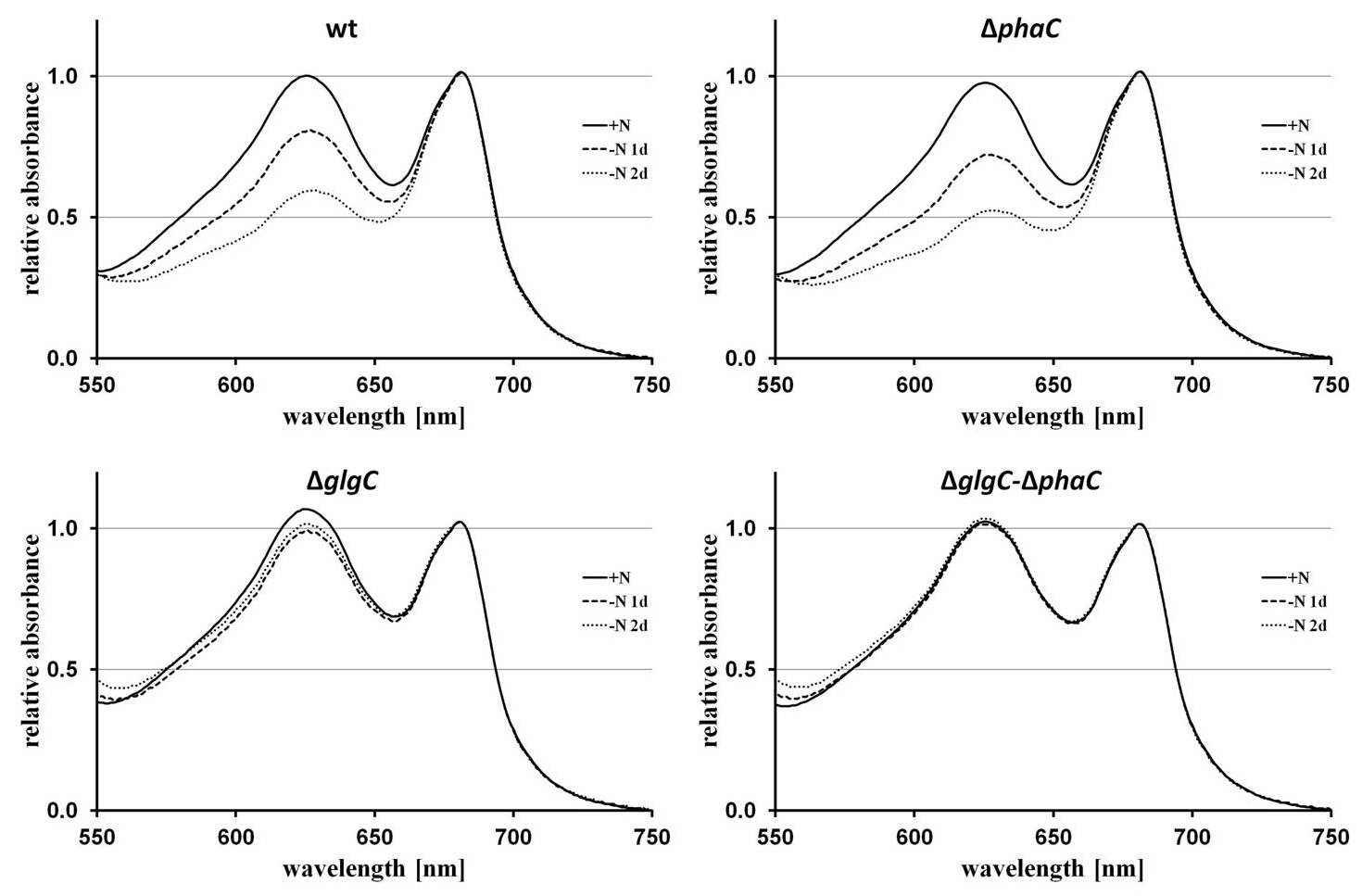
**

**Figure S4: Progression of nitrogen chlorosis by whole-cell absorbance spectra.** The progression of nitrogen chlorosis of wild type (wt) and of mutants defective in glycogen synthesis (Δ*glgC*), PHB synthesis (Δ*phaC*), or both carbon-polymer syntheses (Δ*glgC*/Δ*phaC*) were recorded by whole-cell absorbance spectra using a Specord200 plus (Jena Analytics). The reduction of the phycocyanin peak at 625 nm indicates the primary progression of nitrogen chlorosis as a short-term response to nitrogen depletion (0d, 1d, 2d). The spectra were normalized to the Chlorophyll a peak.
